# Feel-Good Robotics: Requirements on Touch for Embodiment in Assistive Robotics

**DOI:** 10.3389/fnbot.2018.00084

**Published:** 2018-12-11

**Authors:** Philipp Beckerle, Risto Kõiva, Elsa Andrea Kirchner, Robin Bekrater-Bodmann, Strahinja Dosen, Oliver Christ, David A. Abbink, Claudio Castellini, Bigna Lenggenhager

**Affiliations:** ^1^Elastic Lightweight Robotics, Department of Electrical Engineering and Information Technology, Robotics Research Institute, Technische Universität Dortmund, Dortmund, Germany; ^2^Institute for Mechatronic Systems, Mechanical Engineering, Technische Universität Darmstadt, Darmstadt, Germany; ^3^Neuroinformatics Group, Center of Excellence Cognitive Interaction Technology, Bielefeld University, Bielefeld, Germany; ^4^German Research Center for Artificial Intelligence, Robotics Innovation Center, Bremen, Germany; ^5^Robotics Group, University of Bremen, Bremen, Germany; ^6^Department of Cognitive and Clinical Neuroscience, Medical Faculty Mannheim, Central Institute of Mental Health, Heidelberg University, Mannheim, Germany; ^7^Department of Health Science and Technology, Faculty of Medicine, Center for Sensory-Motor Interaction, Aalborg University, Aalborg, Denmark; ^8^School of Applied Psychology, Institute Humans in Complex Systems, University of Applied Sciences and Arts Northwestern Switzerland, Olten, Switzerland; ^9^Delft Haptics Lab, Department of Cognitive Robotics, Faculty 3mE, Delft University of Technology, Delft, Netherlands; ^10^DLR German Aerospace Center, Institute of Robotics and Mechatronics, Oberpfaffenhofen, Germany; ^11^Cognitive Neuropsychology, Department of Psychology, University of Zurich, Zurich, Switzerland

**Keywords:** embodiment, affective touch, social touch, self-touch, human-machine interfaces, tactile feedback, assistive robotics

## Abstract

The feeling of embodiment, i.e., experiencing the body as belonging to oneself and being able to integrate objects into one's bodily self-representation, is a key aspect of human self-consciousness and has been shown to importantly shape human cognition. An extension of such feelings toward robots has been argued as being crucial for assistive technologies aiming at restoring, extending, or simulating sensorimotor functions. Empirical and theoretical work illustrates the importance of sensory feedback for the feeling of embodiment and also immersion; we focus on the the perceptual level of touch and the role of tactile feedback in various assistive robotic devices. We critically review how different facets of tactile perception in humans, i.e., affective, social, and self-touch, might influence embodiment. This is particularly important as current assistive robotic devices – such as prostheses, orthoses, exoskeletons, and devices for teleoperation–often limit touch low-density and spatially constrained haptic feedback, i.e., the mere touch sensation linked to an action. Here, we analyze, discuss, and propose how and to what degree tactile feedback might increase the embodiment of certain robotic devices, e.g., prostheses, and the feeling of immersion in human-robot interaction, e.g., in teleoperation. Based on recent findings from cognitive psychology on interactive processes between touch and embodiment, we discuss technical solutions for specific applications, which might be used to enhance embodiment, and facilitate the study of how embodiment might alter human-robot interactions. We postulate that high-density and large surface sensing and stimulation are required to foster embodiment of such assistive devices.

## 1. Introduction

Due to recent societal trends and technical improvements, assistive robots, i.e., devices that enable or support the performance of a functional task, have gained increased importance in various applications such as prostheses, orthoses, exoskeletons, and devices for teleoperation (Dollar and Herr, 2008; Beckerle et al., 2017; Veneman et al., 2017; Fani et al., 2018). A central issue in assistive robotics is to what extent the device might and should be integrated into the user's bodily self-representation, which has been shown to be highly plastic, e.g., (Moseley et al., 2012). It has previously been argued that particularly robots aiming at restoring, improving, or enhancing human sensorimotor functions might benefit from enhanced embodiment (Pazzaglia and Molinari, 2016; Makin et al., 2017; Niedernhuber et al., 2018). This concerns ownership, i.e., the sensation that an object belongs to the body, as well as agency, i.e., the feeling of being in control of an object's movements (Synofzik et al., 2008). Thus, the crucial question is: how should assistive robots be designed for optimized integration with the user's body?

There has been a keen interest within psychological, neuroscientific, and philosophical studies to investigate to what extent an external object might become part of oneself. Many of these studies focused on the investigation of tool use. Importantly, due to the nature of tool use, this literature largely focuses on motor aspects and visuomotor contingencies. Yet, at least since the seminal study on the rubber hand illusion (Botvinick and Cohen, 1998), research has increasingly investigated the influence of sensory feedback and multisensory processing on the experience of the bodily self. Multisensory integration seems to be a crucial underpinning of the illusion (Bremner and Spence, 2017). Interestingly, a large body of literature suggests that various aspects of touch might differently modulate the feeling of ownership in rubber-hand-illusion-like setups. For example, illusory ownership of a virtual hand can be elicited when participants actively touch the limb (Hara et al., 2015), i.e., during self-touch. Specific, low speed tactile stimulation that activates specific fibers and is associated with positive feelings, i.e., affective touch (Löken et al., 2009), was observed to increase illusory ownership of a rubber hand (Crucianelli et al., 2013, 2017; van Stralen et al., 2014). These results suggest subtle and complex influences of multi-faceted tactile information on the sense of self and the integration of an external object into the person's bodily self-representation. The network of receptors in the human skin is spatially distributed and provides high-density information.

The majority of contemporary tactile feedback techniques, however, focus on low-density and spatially constrained tactile feedback related to active touch of external objects typically through fingers and hand (Antfolk et al., 2013b; Schofield et al., 2014; Svensson et al., 2017; Stephens-Fripp et al., 2018). Yet, this does by no means cover all facets of tactile afferent signals humans typically get from their body when interacting with their environment. Various interaction scenarios also rely on passive touch, i.e., being touched either by one self (self-touch) or by someone else (social/interpersonal touch). Beyond purely functional information about the consequence of a self-generated action, passive and especially social touch might be important in non-verbal communication, for example, in transferring emotional states (Hertenstein et al., 2006, 2009). The required spatial resolution is not yet known, which seems to highlight the limitations of most current technologies, but we think that cutting edge sensory and stimulation devices could help to explore this as argued in detail below.

These facets of touch have preliminarily been investigated in scenarios where they are mediated through a human-machine interface and related to prosthetics, telerobotics, and assistance (Haans and Usselsteijn, 2009; Hara et al., 2015; Huisman, 2017), but are far from being fully understood. Additionally, the applicability of the different facets might depend on the application domain: while it intuitively makes sense that affective tactile feedback might enhance the integration of a prostheses into the bodily self and might foster more natural interpersonal interaction, other assistive devices, e.g., teleoperation in hazardous areas, might not require integration into their user's bodily self, but nevertheless might benefit from more natural feedback to allow for intuitive interaction. Thus, a careful look into the requirements regarding tactile feedback in different domains is indispensable in order to design robots that actually ‘feel good' to their users.

Here, we will discuss to what degree engineering well-integrated tactile feedback could increase the integration of assistive robotic devices such as prostheses (Rosén et al., 2009; Marasco et al., 2011; Bensmaia and Miller, 2014), exoskeletons (Avizzano and Bergamasco, 1999; O'Malley and Gupta, 2008; Frisoli et al., 2009; Ben-Tzvi and Ma, 2015; Mallwitz et al., 2015; Shokur et al., 2016; Planthaber et al., 2018), and telerobotic devices (Gomez-Rodriguez et al., 2011; Gallo et al., 2012; Sengül et al., 2012; Pamungkas and Ward, 2014; Weber and Eichberger, 2015) into their users' body representations. Possibly, increased experience of the bodily self facilitates the use and increases the acceptance and performance of such devices (D'Alonzo and Cipriani, 2012; D'Alonzo et al., 2015; Imaizumi et al., 2016). Furthermore, we consider virtual reality (VR) training approaches as a flexible and low-cost measure (Holden, 2005) to support and enhance users in interacting with assistive robots.

We give theoretical and technical perspectives and suggestions to develop human-robot interactions that embrace affective and social facets of touch as well as self-touch. Therefore, we argue that a shift toward spatially distributed and high-density tactile sensing and stimulation would open up new avenues to empirically investigate user-oriented assistance in terms of applied sciences as well as neuroscientific and psychological research. Finally, the integration of different touch principles might enhance the usability and user experience. We discuss the relations of tactile feedback and perception of robotic devices on a perceptual level as well as how they influence the bodily self-experience and how they are influenced by human-machine interfaces. The subsequent sections discuss potential technologies and applications of high-density bidirectional interfaces with tactile stimulation over large surfaces and their relation to bodily self-experience.

## 2. Technical Solutions for Stimulation and Sensing Devices

We argue that provision of artificial touch feedback enhances bodily self-experience and that the integration of assistive devices into the bodily self improves control (Castellini et al., 2014; Beckerle, 2017). This is highly desirable whenever a human user must learn to use a robotic device as if it was a real extension of his or her body rather than a tool (Hahne et al., 2017). Hence, exploring the functions of the human sense of touch is an important research topic and it still requires substantial technological advancements in several aspects of human-machine interfacing (HMI). From the engineer's point of view, all facets of touch can be enforced via tactile sensing to detect the act of being touched (Dahiya et al., 2010; Zou et al., 2017) and tactile stimulation to elicit the feeling of being touched (Franceschi et al., 2017). To provide all required facets of touch, the HMI must mimic the human sense of touch and implement high sensitivity and distributed sensing and stimulation. Therefore, an ideal HMI enforcing touch comprises an integrated shape-conformable high-density, large-surface tactile stimulator and tactile sensor, with characteristics similar to those of the human skin (Dahiya et al., 2010). First works attempt to provide such HMIs (Kim et al., 2014), but many open research questions remain as outlined in the remainder. Interestingly, robotics research already starts to use whole-body artificial skin to endow humanoid robots with more human-like body experience, which is also used for control purposes, i.e., implementing a safety margin around the robot or supporting it to reach objects (Roncone et al., 2016).

### 2.1. Tactile Stimulation

Tactile stimulation is mainly applied to restore or transmit tactile feedback to a human. Methods for restoring tactile feedback have, for instance, been investigated in prosthetics (Li et al., 2017). However, the focus in previous work has been on providing active touch to improve manipulation or grasping – for example, using force feedback to allow manipulation of delicate objects. So far, affective and social touch have essentially not been considered. Tactile sensations can be elicited using invasive (Raspopovic et al., 2014) and non-invasive (Li et al., 2017) electrical stimulation to depolarize cutaneous afferents or by mechanical stimulation, e.g., vibration motors, force, and torque applicators (Schofield et al., 2014), to directly activate skin mechanoreceptors. However, most of the presented feedback systems are rather simple and include a single stimulator delivering stimuli related to one variable of the assistive robot only, e.g., grasping force of a prosthesis and/or hand aperture (Antfolk et al., 2013a). A few multichannel interfaces have been presented recently, coding feedback information through careful positioning of the stimulators (spatial coding) or using a combination of spatial and parameter modulation (mixed coding) (Dosen et al., 2017; Strbac et al., 2017).

Human-to-human social and affective touch is typically delivered in the form of distributed, non-stationary pressure patterns, e.g., a handshake, and/or gentle motions across the skin, e.g., a caress. To realistically emulate this type of interaction, a specific tactile interface is required, providing high-density stimulation through a dense network of spatially distributed stimulation channels, potentially covering large areas of the user's limb. Electrotactile stimulation is particularly suitable for this application due to its compactness, low power consumption, and rather immediate activation of cutaneous afferents. However, it needs to be considered that electrical stimulation requires calibration as the elicited sensations depend on body location. In addition, if not properly applied, the stimulation can be uncomfortable. Recently, flexible matrix electrodes for electrotactile stimulation have been presented and tested (Štrbac et al., 2016; Franceschi et al., 2017). The electrodes can be printed with a desired distribution and density of stimulation points.

### 2.2. Tactile Sensing

Flexible sensors providing distributed tactile force measurement (artificial skins) have already been presented (Kim et al., 2014; Büscher et al., 2015) and are potentially able to capture the complex mechanical interaction characteristics of social and affective touch. In order to endow an anthropomorphic robotic system such as a hand prosthesis with artificial touch, the tactile sensors need to cover a two-dimensionally curved surface (Kim et al., 2014), while maintaining high resolution and sensitivity. First promising attempts in this direction have been made by combining conductive elastomers with molded-interconnect-devices (MID) (Kõiva et al., 2013). The sensors also need to withstand the wear typical of affective touch situations such as petting and stroking, where the surfaces are slid against each other. Overall, the maintenance cycles, if any, need to be wide apart for the technical systems, for them to be of additional benefit for the user. Many current tactile sensor developments capture the forces normal to the surface; we rather argue that, for affective touch sensing, capturing shear forces is crucial, as affective touch often involves sliding. To the best of our knowledge, no single sensor development exists at present day that achieves all the desired features in one – normal and shear force sensing, high spatial resolution and sensitivity and capability of covering curved surfaces, while being robust enough to be used outside confined laboratory setups.

### 2.3. Integration of Sensing and Stimulation

Tactile sensing and stimulation need to be properly integrated. The data measured by the sensor need to be coded into stimulation profiles delivered by the stimulator. A particular challenge is to accommodate the abundance of tactile data (spatially distributed and high-density). Recently, a prototype system has been demonstrated for transmitting tactile information captured by an artificial skin sensor (64 taxels, i.e., tactile pixels) to a human participant using electrotactile stimulation through a matrix of 32 pads arranged around the forearm. As there were more taxels than pads, the information from several neighboring taxels was fused and mapped to a single pad. Despite using this compromise to cope with current technical limitations, the experimental results showed that human users could recognize a range of shapes (letters, geometries, and lines), retrace the exact trajectory, and guess the direction of the movement of the tactile stimulus (Franceschi et al., 2017). This is an encouraging result for the prospects of inducing and restoring affective and social touch: ideally, repeated, coherent, and discernible tactile stimuli patterns ‘perceived'by the robot could be forwarded to the human via biologically plausible mapping of tactile stimulation. What this mapping should look like, as well as the desirable nature of psychophysical properties of high-density stimulation, are still open questions. Moreover, since a tactile sensation is often associated with a characteristic thermal sensation, e.g., coldness for metal surfaces, warmth for human touch, first studies investigate multimodal feedback including thermal stimulation (Gallo et al., 2012; Pacchierotti et al., 2017).

## 3. Applications in Assistive Robotics

We believe that the ability to control an assistive robotic device is distinctly influencing its usage, acceptance, and integration into the user's bodily self. Moreover, corresponding sensory feedback, as a main part of the sensory-motor loop, is highly relevant for a person to perceive a body part as belonging to oneself. This section discusses how high-density tactile feedback does or could potentially improve different applications of assistive robotics organized by the relevant facets of touch.

### 3.1. Active Touch

Large-surface tactile sensing is an important aspect of modern robotics. Although grippers with high-resolution sensors are able to manipulate and recognize objects only by tactile guidance (Aggarwal et al., 2015), this data are typically used only by the robot control algorithms, but not provided to the human user. Directly conveying active touch could enhance the operator's control over the system and understanding of the environment. Similarly, exoskeletons that assist patients with disabilities are often missing tactile feedback, simplify it distinctly, or make use of sensory substitution, e.g., substituting tactile by vibrotactile feedback (Shokur et al., 2016). Certain sensory substitution approaches try to provide multimodal tactile feedback, e.g., including pressure, vibration, shear force, and temperature (Kim et al., 2010). Such multimodal stimulation could reestablish complex active touch sensations in healthy individuals and patients with somatosensory deprivation by either providing somatotopically matching haptic stimulation (Kim et al., 2010) or by stimulating uneffected body parts with unrestricted sensation (Meek et al., 1989). We argue, that especially for the latter case high-density and large-surface interfaces could enhance touch sensation by possibly enforcing sensory remapping. Natural touch sensation would not only improve the overall experience, but could further enhance learning of simple (Bark et al., 2015) as well as complex motor behavior (Sigrist et al., 2015). This is especially true when combined with VR-based training scenarios that are currently often lacking tactile feedback as well (Weiss et al., 2006; Bovet et al., 2018).

An enhancement of control is even more important for assistive devices that replace a body part, such as prostheses. Here, spatially distributed high-density tactile feedback could provide the missing tactile sensation and allow for an integrated processing of visual and somatosensory feedback to restore the user's feeling of agency and the perceived integrity of the body as investigations with rather simple vibrotactile feedback suggest (Prewett et al., 2012; Witteveen et al., 2015). It has been shown that amputees perceive a varying degree of ownership for their prosthetic devices (Kern et al., 2009), which has been proposed to be an important factor for prosthesis acceptance (Ehrsson et al., 2008). Touch might play a key role in this process: recent studies show that synchronous touches applied to both the residual limb and a prosthetic glove (Ehrsson et al., 2008) and physiologically appropriate cutaneous feedback (Marasco et al., 2011) is capable to induce vivid ownership sensations for a prosthesis, which not only becomes a tool, but an integrated part of the body (Graczyk et al., 2018). It turns out that the synchrony of stimulation and context (Rohde et al., 2011; Bekrater-Bodmann et al., 2012) as well as synchrony of multimodal sensory input (Choi et al., 2016) is of key importance.

### 3.2. Passive Touch

Passive touch is highly relevant for various domains of assistive robotics. Examples are telerobotics, e.g., the visual sight is very limited or simply not given under water, or during everyday use of robotic prostheses, e.g., when being touched in the dark or outside the field of view. Thus, an artificial skin to detect contacts with the environment should cover the whole surface of the robotic device. Such an approach could enhance the robots' and thus also the users' or teleoperators' insight into the actual environmental situation. Therefore, the HMI might cover large areas of the user's body to haptically mirror the robot's surface to the user. This could foster the integration of a teleoperated robot's body into the operator's self-representation for higher immersion and thus better control and could contribute to the restoration of perceived body integrity of individuals using prostheses. Despite technical feasibility, HMI design would then also need to consider cognitive burden of the human processing the various feedback (Beckerle et al., 2017).

### 3.3. Affective and Social Touch

Providing feedback also affectively might contribute to the integration of robotic devices into the bodily self-representation, similarly to recent observations in the rubber hand illusion paradigm (Crucianelli et al., 2013, 2017; van Stralen et al., 2014). The application of affective touch feedback in prosthesis users is promising, since the skin of the residual limb potentially remains sensitive for those signals. Hence, we expect prosthetic applications to strongly benefit from high-density and large surface feedback.

Social touch is at the basis of daily interpersonal interaction and non-verbal communication and first tailored haptic stimulators are under development (Culbertson et al., 2018). Since social touch can support human-human interaction, it might be helpful in robot-assisted caregiving contexts as well as in enhancing the acceptance of prosthetic devices, particularly for the upper extremity, as prior findings show that successful prosthesis use in terms of satisfaction and frequency is positively associated with social integration (Ham and Cotton, 2013).

Today, VR-based training scenarios help to improve user control in teleoperation and exoskeleton mediated assistance (Lugo-Villeda et al., 2009; Folgheraiter et al., 2012; Fani et al., 2018). While haptic guidance supports the users' trajectory tracking capabilities (Lugo-Villeda et al., 2009), the support of a therapist could be mimicked by introducing the feeling of her or him touching a patient to prevent unwanted movements. Such a social touch must be different with respect to the forces introduced to guide the patient and could further improve the support of patients especially in VR-based therapy applications.

### 3.4. Self-Touch

Self-touch is often neglected, but important in order to establish and maintain our own body representation (Bremner and Spence, 2017). Moreover, this kind of touch has been shown to increase body ownership (Hara et al., 2015) and first preliminary studies have extended such scenarios to rather complex situations of self-touch (Dieguez et al., 2009; Huynh et al., 2018). Research on embodiment in VR also shows that correct self-contact is more important for the feeling of embodiment than the mapping of movements (Bovet et al., 2018). Yet, tactile feedback is often limited to the hand or body parts, which was shown to constrain the implementation of passive self-touch in VR (Bovet et al., 2018).

## 4. Conclusion and Outlook

This paper points out the importance of various facets if tactile feedback for the embodiment of assistive robotic devices. Notably, self-touch, affective touch, and social touch should be considered as they modulate embodiment on different levels and ultimately concern psychosocial factors that determine how the device will be used in real life. To communicate this diverse tactile information between human and machine, sensing and stimulation needs to be spatially distributed and to be provided with high-density. Our review shows that several promising technologies such as flexible tactile sensors or electrotactile stimulation exist, but need to be improved and integrated into one interface. The design of the non-stationary and complex stimulation patterns to provide the actual tactile feedback might take human-human interaction as an example.

Finally, we expect that versatile and realistic tactile feedback covering the different facets of touch will enhance the usability and the user experience of assistive robots. We advocate the inclusion of self-touch, affective touch, and social touch to provide interfaces that approximate the capabilities of human skin. We recommend fundamental research toward a practical framework for psychology of tool use that can provide guidelines toward engineering for embodiment. A tight collaboration of engineering and psychology will be needed to devise experimental protocols as well as day-to-day interaction strategies between machines and humans. Advanced robotic touch technology embedded into robotic artifacts will likely be the main tool to enforce a real synergy with users.

## Author Contributions

PB coordinated the development of the paper and the integration of individual contributions. All authors contributed content, perspectives, and references as well as discussed and revised the manuscript.

### Conflict of Interest Statement

The authors declare that the research was conducted in the absence of any commercial or financial relationships that could be construed as a potential conflict of interest.
